# *In Vitro* Measles Virus Infection of Human Lymphocyte Subsets Demonstrates High Susceptibility and Permissiveness of both Naive and Memory B Cells

**DOI:** 10.1128/JVI.00131-18

**Published:** 2018-03-28

**Authors:** Brigitta M. Laksono, Christina Grosserichter-Wagener, Rory D. de Vries, Simone A. G. Langeveld, Maarten D. Brem, Jacques J. M. van Dongen, Peter D. Katsikis, Marion P. G. Koopmans, Menno C. van Zelm, Rik L. de Swart

**Affiliations:** a Department of Viroscience, Postgraduate School of Molecular Medicine, Erasmus MC, University Medical Centre Rotterdam, Rotterdam, the Netherlands; b Department of Immunology, Postgraduate School of Molecular Medicine, Erasmus MC, University Medical Centre Rotterdam, Rotterdam, the Netherlands; University of Kentucky College of Medicine

**Keywords:** measles, tropism, immunosuppression, immune amnesia, viral pathogenesis

## Abstract

Measles is characterized by a transient immune suppression, leading to an increased risk of opportunistic infections. Measles virus (MV) infection of immune cells is mediated by the cellular receptor CD150, expressed by subsets of lymphocytes, dendritic cells, macrophages, and thymocytes. Previous studies showed that human and nonhuman primate memory T cells express higher levels of CD150 than naive cells and are more susceptible to MV infection. However, limited information is available about the CD150 expression and relative susceptibility to MV infection of B-cell subsets. In this study, we assessed the susceptibility and permissiveness of naive and memory T- and B-cell subsets from human peripheral blood or tonsils to *in vitro* MV infection. Our study demonstrates that naive and memory B cells express CD150, but at lower frequencies than memory T cells. Nevertheless, both naive and memory B cells proved to be highly permissive to MV infection. Furthermore, we assessed the susceptibility and permissiveness of various functionally distinct T and B cells, such as helper T (T_H_) cell subsets and IgG- and IgA-positive memory B cells, in peripheral blood and tonsils. We demonstrated that T_H_1T_H_17 cells and plasma and germinal center B cells were the subsets most susceptible and permissive to MV infection. Our study suggests that both naive and memory B cells, along with several other antigen-experienced lymphocytes, are important target cells of MV infection. Depletion of these cells potentially contributes to the pathogenesis of measles immune suppression.

**IMPORTANCE** Measles is associated with immune suppression and is often complicated by bacterial pneumonia, otitis media, or gastroenteritis. Measles virus infects antigen-presenting cells and T and B cells, and depletion of these cells may contribute to lymphopenia and immune suppression. Measles has been associated with follicular exhaustion in lymphoid tissues in humans and nonhuman primates, emphasizing the importance of MV infection of B cells *in vivo*. However, information on the relative susceptibility of B-cell subsets is scarce. Here, we compared the susceptibility and permissiveness to *in vitro* MV infection of human naive and memory T- and B-cell subsets isolated from peripheral blood or tonsils. Our results demonstrate that both naive and memory B cells are more permissive to MV infection than T cells. The highest infection levels were detected in plasma cells and germinal center B cells, suggesting that infection and depletion of these populations contribute to reduced host resistance.

## INTRODUCTION

Measles virus (MV) is an enveloped virus with a single-stranded negative-sense RNA genome that belongs to the family Paramyxoviridae, genus Morbillivirus. This highly contagious virus is transmitted via the respiratory route and causes systemic disease in humans and nonhuman primates (NHPs). Measles is characterized by fever, cough, and skin rash (1, 2). The hallmark of the disease is a transient immune suppression, which leads to increased susceptibility to opportunistic infections (3). Common complications include bacterial pneumonia, otitis media, or diarrhea. Despite the availability of safe and effective live-attenuated vaccines (4), measles remains an important cause of global childhood mortality. In 2016, more than 85,000 people, mostly children below the age of 5 years, died due to measles and its sequelae (5).

Measles virus is an intrinsically dual-tropic virus that uses two different cellular entry receptors (6). Infection of immune cells is mediated by CD150, a member of the signaling lymphocytic activation molecule (SLAM) family. CD150 is expressed by subsets of T and B cells, dendritic cells (DCs), macrophages, thymocytes, and hematopoietic stem cells and mediates the entry and dissemination of MV (7–9). Infection of epithelial cells is mediated by nectin-4 and is considered of crucial importance for virus transmission (10, 11).

Measles is associated not only with immune suppression but also with a paradoxical immune activation (12, 13). Uncomplicated measles in children with a functional immune system is typically a self-limiting disease, and recovery is attributed to strong MV-specific immune responses that confer lifelong immunity. Lymphopenia is common during the acute stage of measles and lasts for about 1 week, whereas immune suppression can last for several weeks, months, or even years (3, 14, 15). Shortly after clearance of MV-infected cells by the host cellular immune response, the numbers of circulating lymphocytes return to normal. On the basis of these observations, a model was postulated to explain measles-induced immune suppression: preexisting CD150^+^ memory lymphocytes are depleted during MV infection, resulting in a temporary immunological amnesia. The depletion of CD150^+^ cells is masked by the expansion of MV-specific and bystander lymphocytes (14, 16).

MV also infects and replicates in B cells (17–22). Studies in humans and NHPs have shown that MV infects B-cell follicles in lymphoid tissues and causes follicular exhaustion during and shortly after the peak of viremia in humans and macaques (14, 17, 18, 23, 24). For a successful viral infection, cells must be accessible, susceptible, and permissive. Susceptible cells express receptors on the cell surface that allow entry of the virus, while permissive cells can support viral replication (25). Multiple subsets of B cells are known to express and upregulate the expression of CD150 on their surfaces upon maturation, development, and differentiation (14, 26), but their relative susceptibility to MV has not yet been described. Given the importance of CD150 in MV infection and the lack of a complete understanding of the interaction between MV and specific lymphocyte subsets, we investigated the relative susceptibility of human peripheral blood and tonsillar T- and B-cell subsets to *in vitro* MV infection. We demonstrate that both naive and memory B cells are susceptible and permissive to *in vitro* MV infection.

## RESULTS

### Lower frequency of CD150^+^ cells in peripheral blood B cells than in T cells.

**FIG 1:**
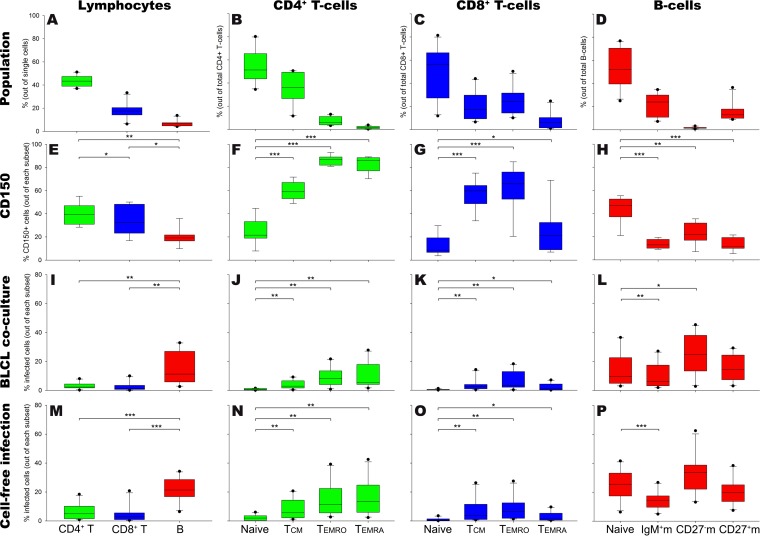
Susceptibility and permissiveness of human peripheral blood T- and B-cell subsets to *in vitro* MV infection. Human PBMC (*n* = 10 donors) were gated into CD4^+^ and CD8^+^ T cells and B cells and further subtyped into naive and memory cells. (A to D) Frequencies of T cells, B cells, and their subsets in blood PBMC; (E to H) frequencies of CD150^+^ cells within T- and B-cell subsets; (I to L) frequencies of MV-infected PBMC following 30 h of coculture with autologous MV-infected BLCL; (M to P) frequencies of MV-infected PBMC following cell-free inoculation. IgM^+^m, IgM^+^ memory B cells; CD27^−^m, CD27^−^ memory B cells; CD27^+^m, CD27^+^ memory B cells. Data are presented as box plots. *, *P* < 0.05; **, *P* < 0.01; ***, *P* ≤ 0.001.

We determined the frequencies of T and B cells and their subsets (as defined in Table 1) in peripheral blood mononuclear cells (PBMC) of healthy adult donors (Fig. 1A to D), as well as the frequencies of cells expressing CD150 in each of these populations (Fig. 1E to H). Previous studies have shown that CD4^+^ and CD8^+^ memory T cells expressed higher levels of CD150 than their naive counterparts (14, 24). Consistent with these findings, we found that within the CD4^+^ and CD8^+^ T-cell subsets, more memory than naive T cells expressed CD150 (Fig. 1F and G). B cells contained fewer cells that expressed CD150 (Fig. 1E), and, in contrast to T cells, the frequencies of CD150^+^ cells in the naive B-cell subset were significantly higher than those in the memory subsets (Fig. 1H).

**TABLE 1:** Definition of peripheral blood and tonsillar lymphocyte subsets^*a*^

Subset name	Definition
T cells	CD3^+^
CD4^+^ T cells	CD3^+^ TCRγδ^−^ CD4^+^ CD8^−^
Naive cells	CD3^+^ TCRγδ^−^ CD4^+^ CD8^−^ CD45RA^+^ CCR7^+^ CD27^+^
Memory cells	
T_CM_	CD3^+^ TCRγδ^−^ CD4^+^ CD8^−^ CD45RA^−^ CCR7^+^ CD27^+^
T_EMRO_	CD3^+^ TCRγδ^−^ CD4^+^ CD8^−^ CD45RA^−^ CCR7^−^
T_EMRA_	CD3^+^ TCRγδ^−^ CD4^+^ CD8^−^ CD45RA^+^ CCR7^−^
T_FH_	CD3^+^ TCRγδ^−^ CD4^+^ CD8^−^ CD45RA^−^ CXCR5^+^
T_H_1	CD3^+^ CD4^+^ CD8^−^ CD45RA^−^ CD25^low^ CCR6^−^ CXCR3^+^ CCR4^−^
T_H_2	CD3^+^ CD4^+^ CD8^−^ CD45RA^−^ CD25^low^ CCR6^−^ CXCR3^−^ CCR4^+^
T_H_1T_H_17	CD3^+^ CD4^+^ CD8^−^ CD45RA^−^ CD25^low^ CCR6^+^ CXCR3^+^ CCR4^−^
T_H_17	CD3^+^ CD4^+^ CD8^−^ CD45RA^−^ CD25^low^ CCR6^+^ CXCR3^−^ CCR4^+^
Treg	CD3^+^ CD4^+^ CD8^−^ CD45RA^−^ CD25^high^ CD127^−/low^
CD8^+^ T cells	CD3^+^ TCRγδ^−^ CD4^−^ CD8^+^
Naive cells	CD3^+^ TCRγδ^−^ CD4^−^ CD8^+^ CD45RA^+^ CCR7^+^ CD27^+^
Memory cells	
T_CM_	CD3^+^ TCRγδ^−^ CD4^−^ CD8^+^ CD45RA^−^ CCR7^+^ CD27^+^
T_EMRO_	CD3^+^ TCRγδ^−^ CD4^−^ CD8^+^ CD45RA^−^ CCR7^−^
T_EMRA_	CD3^+^ TCRγδ^−^ CD4^−^ CD8^+^ CD45RA^+^ CCR7^−^
B cells	CD19^+^
Transitional	CD19^+^ CD21^high^ CD24^+^ CD27^−^ CD38^+^
Plasma cells	CD19^+^ CD21^high^ CD24^−^ CD27^+^ CD38^+^
GC cells	CD19^+^ CD21^high^ CD24^−^ CD38^high^
Naive cells	CD19^+^ CD21^high^ CD38^low^ CD27^−^ IgM^+^
Memory cells	
IgM^+^/non-CSM cells	CD19^+^ CD21^high^ CD38^low^ CD27^+^ IgM^+^
IgM-only cells	CD19^+^ CD21^high^ CD38^low^ CD27^+^ IgM^+^ IgD^−^
Natural effector	CD19^+^ CD21^high^ CD38^low^ CD27^+^ IgM^+^ IgD^+^
CSM B cells	CD19^+^ CD21^high^ CD38^low^ CD27^−/+^ IgM^−^
IgG^+^ cells	CD19^+^ CD21^high^ CD38^low^ CD27^−/+^ IgM^−^ IgD^−^ IgA^−^ IgG^+^
IgA^+^ cells	CD19^+^ CD21^high^ CD38^low^ CD27^−/+^ IgM^−^ IgD^−^ IgA^+^ IgG^−^

a T_CM_, central memory T cells; T_EMRO_/T_EMRA_, CD45RO^+^/CD45RA^+^ effector memory T cells; T_FH_, follicular helper T cells; T_H_, helper T cells; Treg, regulatory T cells; non-CSM, non-class-switched memory; CSM, class-switched memory; GC, germinal center.

### Higher frequency of MV-infected cells in peripheral blood B cells than in T cells.

Next, we evaluated the permissiveness of the T- and B-cell subsets described above after *in vitro* MV infection. MV dissemination *in vivo* is mostly mediated by direct cell-to-cell transmission of virus. To mimic this process, freshly isolated PBMC (*n* = 6 donors) were cocultured with cells of a rMV^KS^Venus(3)-infected autologous B-lymphoblastoid cell line (BLCL) (27). In these experiments, expression of the fluorescent reporter protein Venus was used to identify MV-infected cells. We validated these experiments with wild-type MV strain MVi/Amsterdam.NLD/19.11 (*n* = 4 donors) and identified the wild-type MV-infected cells using intracellular staining of the virus nucleoprotein (NP). To assess the levels of MV-infected cells from the first round of infection, we determined the percentages of infected cells after 30 h of coculture (Fig. 1I to L). In accordance with the findings of previous studies (14, 24), we found that within the T-cell populations, MV preferentially infected CD4^+^ and CD8^+^ memory T cells (Fig. 1J and K). Surprisingly, despite the lower percentage of CD150-expressing cells, a higher frequency of MV-infected cells was found among B cells than CD4^+^ and CD8^+^ T cells (Fig. 1I). Moreover, in B-cell populations, despite the higher frequency of CD150^+^ cells within the naive cells than within the memory cells, there was no preferential infection of naive B cells, as all subsets were susceptible and permissive to MV infection (Fig. 1L).

Since autologous BLCL were not always available for coculture, we also inoculated the freshly isolated PBMC with cell-free rMV^KS^Venus(3) or wild-type MV strain MVi/Amsterdam.NLD/19.11 at multiplicity of infection (MOI) of 1 in the presence of infection-enhancing lipopeptide (Fig. 1M). The susceptibility and permissiveness patterns of CD4^+^ and CD8^+^ memory T cells and B cells were largely reproduced in this cell-free inoculation (Fig. 1N to P).

### MV preferentially infects tonsillar B cells.

**FIG 2:**
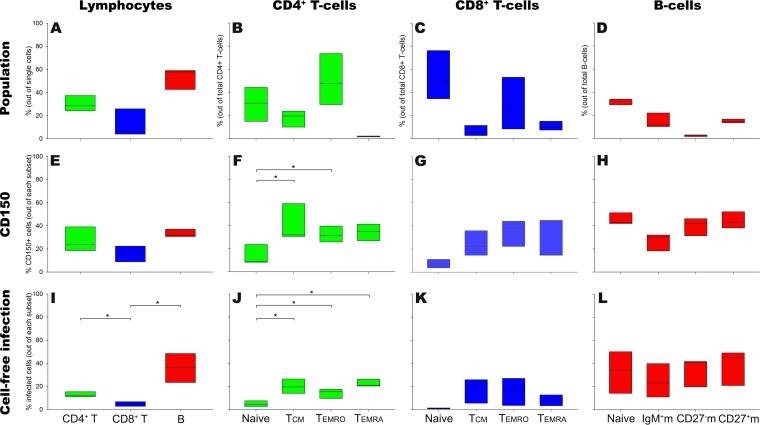
Susceptibility and permissiveness of human tonsillar T- and B-cell subsets to *in vitro* MV infection. Human tonsillar lymphocytes (*n* = 3 donors) were gated into CD4^+^ and CD8^+^ T cells and B cells and further subtyped into naive and memory cells. (A to D) Frequencies of human tonsillar T cells, B cells, and their subsets; (E to H) frequencies of CD150^+^ cells within T- and B-cell subsets; (I to L) frequencies of MV-infected tonsillar lymphocytes following cell-free inoculation. IgM^+^m, IgM^+^ memory B cells; CD27^−^m, CD27^−^ memory B cells; CD27^+^m, CD27^+^ memory B cells. Data are presented as box plots. *, *P* < 0.05.

PBMC contain a relatively low percentage of B cells (4 to 14%; Fig. 1A). Some B-cell subsets, including germinal center (GC) B cells and plasma cells, do not circulate at substantial levels. Moreover, MV replicates first and foremost in lymphoid tissues *in vivo*. Thus, we validated our findings using single-cell suspensions from human tonsils, of which 42 to 59% consisted of B cells (Fig. 2A). Lymphocyte subsets (Fig. 2B to D) and the frequencies of CD150^+^ cells (Fig. 2E to H) were defined and determined as described above for PBMC. CD150^+^ cells were more frequent within the tonsillar memory CD4^+^ and CD8^+^ T cells than within their naive counterparts (Fig. 2F to G). Between the tonsillar naive and memory B cells, the fractions of CD150^+^ cells were not significantly different (Fig. 2H). Upon *in vitro* cell-free inoculation with MV, B cells had a higher frequency of MV-infected cells than T cells (Fig. 2I). Still in accordance with previous findings (14), tonsillar memory T cells within both the CD4^+^ and CD8^+^ subsets were preferentially infected compared to naive T cells (Fig. 2J to K). Within the tonsillar B-cell subsets, this preferential infection of naive or memory cells was not observed (Fig. 2L).

### Functionally distinct T- and B-cell subsets have different MV infection levels.

**FIG 3:**
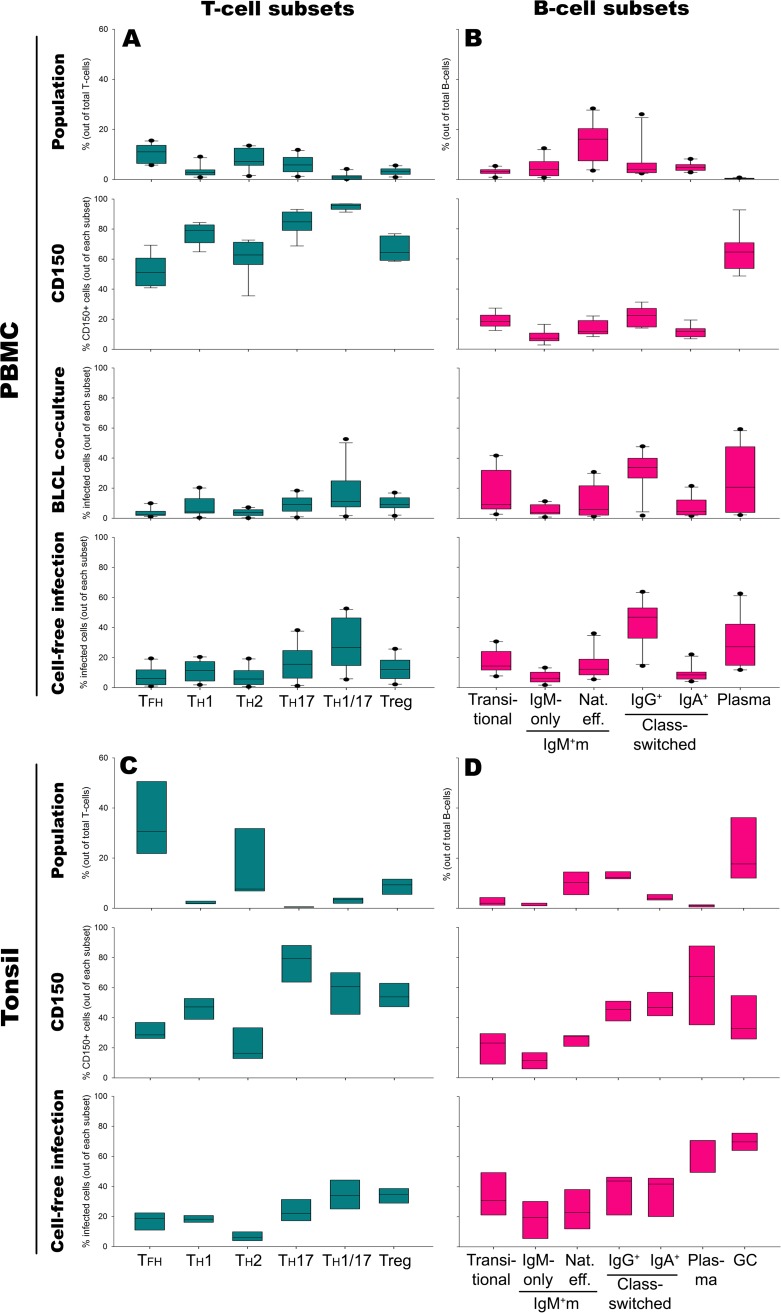
Susceptibility and permissiveness of specific effector T- and B-cells in peripheral blood (*n* = 10 donors) and tonsils (*n* = 3). The frequencies of T- and B-cell effector subsets in blood PBMC (A and B) or tonsils (C and D), the frequencies of CD150^+^ cells within the subsets, and the frequencies of MV-infected cells following either 30 h of coculture with autologous MV-producing BLCL or cell-free inoculation of MV are shown. Nat. eff., natural effector; IgM^+^m, IgM^+^ memory B cells. Data are presented as box plots.

Several T- and B-cell subsets have been described to have distinct functions in the immune response to pathogens. We determined the frequencies of CD150^+^ cells within a number of circulating CD4^+^ T-cell subsets, follicular helper T cells (T_FH_ cells), helper T cells (T_H_1, T_H_2, T_H_17, and T_H_1T_H_17 cells), and regulatory T cells (Treg cells) (Fig. 3A), and assessed their MV infection levels. T_FH_ cells are a specific effector helper T-cell subset responsible for the interaction with B cells in the GC. This population resides in the lymph nodes, although a fraction of the cells could be found circulating in blood as well (Fig. 3A). T_FH_ cells express various SLAM family members (9). In this study, we observed that a median of 52% of circulating T_FH_ cells expressed CD150, but only a median of 2 or 6% of T_FH_ cells was infected following BLCL coculture or cell-free infection, respectively. Other important effector subsets include the T_H_1 and T_H_2 cells. In accordance with the findings of previous studies reporting a higher CD150 mRNA level in T_H_1 cells than in T_H_2 cells (28), we found more CD150^+^ T_H_1 cells (median, 78%) than CD150^+^ T_H_2 cells (median, 61%). Despite these high percentages of susceptible cells, infection levels were consistently below 20% of the respective subsets. Treg cells showed a similar pattern with a median of 65% CD150^+^ cells but less than 25% MV-infected cells. The CCR6^+^ T_H_17 and T_H_1T_H_17 cell subsets contained the highest frequencies of CD150^+^ and MV-infected cells within the T-cell compartment.

We next assessed the expression of CD150 on further defined circulating B-cell subsets, transitional, non-class-switched IgM-positive (IgM^+^) memory (IgM-only and natural effector B-cell) subsets, class-switched CD27^−^ IgM-negative (IgM^−^) and CD27^+^ IgM^−^ memory (IgG^+^ and IgA^+^) subsets, and plasma cells, and also their MV infection levels. CD150^+^ and MV-infected cells were found at high frequencies among transitional, IgG^+^ memory, and plasma cells (Fig. 3B).

Studies in macaques inoculated with recombinant MV expressing enhanced green fluorescence protein showed that MV-infected cells migrate from the site of inoculation into the draining lymph nodes and rapidly infect susceptible lymphocytes, resulting in T- and B-cell depletion in the lymphoid tissues (14). Nonetheless, the susceptible subsets of these tissue lymphocytes have been defined only rudimentarily. To gain more insight into this, we investigated specific human tonsillar T- and B-cell subsets for their frequencies of CD150^+^ and MV-infected cells. Similar to the circulating T-cell population, the highest frequencies of CD150^+^ and MV-infected cells were found among the T_H_1T_H_17 cell subset. Surprisingly, tonsillar Treg cells had a lower frequency of CD150^+^ cells but a higher frequency of MV-infected cells than their peripheral blood equivalents (Fig. 3C). Among tonsillar B-cell subsets, IgG^+^ and IgA^+^ memory cells had comparable frequencies of CD150^+^ cells and MV-infected cells. A specific B-cell subset known as GC B cells was present in high numbers in lymphoid tissues, but not in the circulation (29). Although the frequency of CD150^+^ cells in this subset was relatively low, they had the highest frequency of MV-infected cells (Fig. 3D).

### MV-infected B cells produce infectious virus.

**FIG 4:**
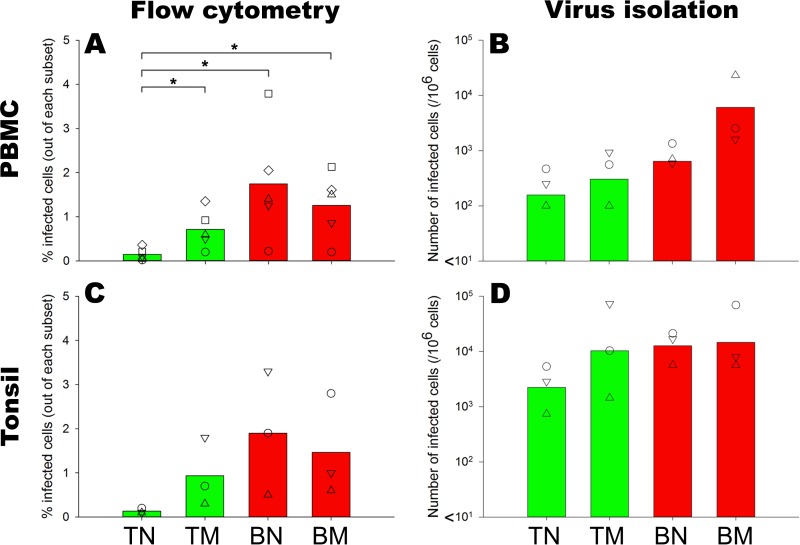
Susceptibility and permissiveness (determined by flow cytometry) and number of productive infected sorted peripheral blood (A and B) or tonsillar (C and D) naive and memory CD4^+^ T and B cells (determined by virus isolation). Sorted naive and memory B cells retained their susceptibility to MV infection, despite the absence of other lymphocytes, suggesting that this is an intrinsic characteristic of the cells. When these infected cells were cocultured with susceptible cells, it resulted in a high number of newly infected cells. TN, CD4^+^ naive T cells; TM, CD4^+^ memory T cells; BN, naive B cells; BM, memory B cells. *, *P* < 0.05.

Infection of cells with a Venus-expressing recombinant MV can be assessed by measuring the percentage of Venus-positive (Venus^+^) cells or by determining the number of infected cells capable of transmitting the virus to new susceptible cells. We obtained purified naive and memory CD4^+^ T- and B-cell subsets from human PBMC by fluorescence-activated cell sorting (FACS) (Table 2) and inoculated each subset with rMV^KS^Venus(3) at an MOI of 3 in the presence of infection-enhancing lipopeptide. After 24 h, the infection percentages in the sorted subsets were determined by flow cytometry. In accordance with previous findings, the frequency of infected cells in CD4^+^ T-cell subsets, defined as Venus^+^ cells, was found to be lower in sorted naive T cells than in the memory cells. Among the B-cell subsets, the frequency of infected cells in sorted naive cells was comparable to that in the memory cells (Fig. 4A).

**TABLE 2:** Definition of sorted peripheral blood and tonsillar lymphocyte subsets^*a*^

Subset name	Definition
CD4^+^ TN	CD3^+^ CD19^−^ CD4^+^ CD8^−^ CD45RO^−^ CCR7^+^
CD4^+^ TM	CD3^+^ CD19^−^ CD4^+^ CD8^−^ CD45RO^+^ CCR7^−^
BN	CD19^+^ CD38^low^ CD27^−^ IgM^+^
BM	CD19^+^ CD38^low^ CD27^+^ IgM^−^

a TN, naive T cells; TM, memory T cells; BN, naive B cells; BM, memory B cells.

To assess the capability of MV-infected cells to produce new infectious virus particles, we assessed the numbers of MV-infected sorted cells that can infect new, susceptible cells. MV-infected sorted naive T cells, being the least susceptible subset to MV infection, gave rise to a lower number of newly infected cells than memory T cells. Surprisingly, despite the comparable susceptibility of naive and memory B cells to MV infection, MV-infected sorted memory B cells generated a higher frequency of infected cells than naive cells (Fig. 4B). We verified these findings in sorted tonsillar lymphocytes. Naive T cells were less susceptible to MV infection and had a lower frequency of productive infected cells than memory T cells. In accordance with their peripheral blood counterparts, tonsillar naive and memory B cells were highly susceptible to MV infection (Fig. 4C). However, in contrast to circulating B cells, the number of productive infected tonsillar naive B cells was as high as that of memory B cells (Fig. 4D). Altogether, these findings demonstrate that both naive and memory B cells are susceptible and permissive to MV infection, and their infection leads to dissemination of infectious virus.

## DISCUSSION

In this study, we have directly compared the susceptibility and permissiveness of human lymphocyte subsets to *in vitro* MV infection. Our results were compliant with previous observations, in which memory T cells were more susceptible than their naive counterparts. Although the frequencies of CD150-expressing B cells were lower than (in blood) or comparable to (in tonsils) those of T cells, both naive and memory B cells showed substantially higher *in vitro* MV infection levels, suggesting that B cells are more permissive to MV infection than T cells. Direct comparison of functionally distinct T- and B-cell subsets identified T_H_1T_H_17 cells, plasma cells, and GC B cells to be subsets highly susceptible and permissive to MV infection.

Previous studies have identified alveolar macrophages and DCs to be the initial target cells of MV. These cells migrate to the draining lymphoid tissues, where they come in contact with susceptible CD150^+^ T and B cells (30), resulting in cell-associated viremia and viral dissemination to all lymphoid tissues. To understand the impact of MV infection on the immune system, it is important to assess differences in the susceptibility and permissiveness of lymphocyte subsets. Although several studies have shown the higher susceptibility of memory T cells than naive T cells, information on differences in susceptibility and the permissiveness of B-cell subsets was limited.

In this study, we included naive and memory T-cell subsets as internal controls to validate our *in vitro* infection model. After we confirmed that memory T cells have higher susceptibility than naive T cells to MV infection, as previously described, we expanded our observations to include T-cell subsets with specific effector functions, since their depletion may contribute to measles immune suppression. Among these are T_FH_ cells, which constitute a major fraction of the lymphoid tissue T-cell population and secrete cytokines that are critical for induction of B-cell proliferation, isotype switching, and antibody secretion. Other helper T (T_H_) cell subsets, such as T_H_1, T_H_2, T_H_17, T_H_1T_H_17, and Treg cells, have also been reported to play crucial effector roles during the immune responses against various pathogens (31, 32). Interestingly, we observed a relatively high susceptibility and permissiveness of T_H_17 and T_H_1T_H_17 cell subsets. T_H_17 cells have been reported to disrupt the blood-brain barrier (BBB) and promote the recruitment of white blood cells into the central nervous system (CNS), while T_H_1T_H_17 cells were demonstrated to migrate across the BBB (32). Therefore, it may be speculated that these cells could play a role in the occasional spread of MV infection into the central nervous system.

In the lymph nodes, activated B cells interact with antigen-specific T_H_ and T_FH_ cells, resulting in the generation of GC, in which the B cells proliferate and undergo somatic hypermutation and class-switch recombination. The surviving B cells differentiate into either memory B cells or plasma cells and leave the GC. Memory B cells circulate as resting B cells until reactivation (33, 34). We observed that plasma cells, class-switched memory B cells, and GC cells were highly susceptible and permissive to *in vitro* MV infection. The susceptibility of GC B cells to MV infection was highlighted in clinical case studies in the 1970s and 1980s, which reported dramatic improvements in patients with Burkitt's and Hodgkin's lymphomas after infection with wild-type MV. These diseases are associated with Epstein-Barr virus (EBV) infection (35–37), and some studies have strongly suggested that the malignant cells derive from GC B cells (38, 39). The lymphotropic nature of wild-type MV, together with the high susceptibility of GC B cells to MV infection, may thus have contributed to the tumor regression in these patients. The susceptibility of these malignant cells is also underpinned by the fact that EBV-transformed BLCL are highly susceptible to MV infection *in vitro* and have been used for virus production and reisolation purposes (40). Given that B-cell follicles were depleted in the lymph nodes of humans and NHPs infected with MV, we speculate that normal human GC B cells are also highly susceptible and permissive *in vivo*. The depletion of such an important subset may hinder the development of plasma and memory B cells. This, in combination with the partial depletion of preexisting CD150^+^ plasma and class-switched memory B cells, may result in the restriction of humoral immune responses to other pathogens after measles and thus contribute to the increased risk of opportunistic infections and other measles-associated complications.

Differences in the efficiency of *in vitro* MV infection of naive and memory T cells can largely be explained by their susceptibility, as determined by differences in the frequency of cells expressing CD150. Interestingly, several B-cell subsets, including IgG^+^ memory and GC B cells, showed high MV infection levels, even though moderate or low frequencies of these expressed CD150. This suggests that differences in the efficiency of MV infection in lymphocyte subsets are determined not solely by the expression of CD150 but also by differences in host permissiveness to viral infection. We speculate that these differences may be related either to differences in host metabolic activity or to their innate responses to infection. Further investigations are required to validate these observations. In this study, we observed on several occasions that the percentage of MV-infected cells was higher than the percentage of CD150^+^ cells within a specific lymphocyte subset. We have previously observed that CD150 expression by PBMC is usually detected as a continuous gradient, instead of two clearly separated populations of CD150^−^ and CD150^+^ cells (41). It should be noted that, in the current study, the cutoff for positivity in the CD150 staining was determined by comparison to an isotype control staining, and thresholds were always set conservatively. Thus, although the cells that were defined as CD150^+^ cells are truly positive for CD150 expression, it cannot be concluded that the remaining cells are all negative for CD150 expression. Some cells with low levels of CD150 expression that did not reach our cutoff for positivity could still be susceptible to MV infection.

The susceptibility and permissiveness of naive and memory T and B cells were still retained when the cells were sorted from the total lymphocyte population, regardless of their origin from blood or tonsil. This suggests that the susceptibility and permissiveness to *in vitro* MV infection is an inherent characteristic of the cells independent of the influence of other cells. We further observed that blood and tonsillar B cells not only were infected but also could give rise to new infected cells. To what extent the infectious virus production by B cells contributes to the systemic dissemination of MV *in vivo* remains to be evaluated.

In conclusion, our study has shown that both naive and memory B cells of peripheral blood and tonsillar origin were susceptible and permissive to MV infection *in vitro* and gave rise to newly infected cells, indicating their role as important target cells for MV. These findings provide new insights into the pathogenesis of measles and measles-induced immune suppression.

## MATERIALS AND METHODS

### Ethics statement.

PBMC were obtained from healthy adult donors, and tonsils were obtained from children undergoing tonsillectomy (Erasmus MC-Sophia Children's Hospital, Rotterdam, the Netherlands). Written informed consent was obtained from the donors (for PBMC) or from both parents (for the tonsils), and the studies were approved by the Medical Ethical Committee of Erasmus MC, Rotterdam, the Netherlands (MEC-2015-0955 and MEC-2013-093). For experiments involving human buffy coats, written informed consent for research use was obtained by the Sanquin Blood Bank.

### Cells.

Human PBMC were isolated from blood (*n* = 10 healthy adult donors) by density gradient centrifugation. BLCL were established by transformation with Epstein-Barr virus (EBV) as previously described (42). BLCL were cultured in RPMI 1640 (Lonza, Belgium) supplemented with 10% fetal bovine serum (FBS; Sigma-Aldrich, USA), 100 IU of penicillin/ml and 100 μg of streptomycin/ml (Lonza, Belgium), and 2 mM l-glutamine (Lonza, Belgium) (R10F medium).

Tonsillar single-cell suspensions were prepared from three different donors as previously described (43). Cells were pelleted at 500 × *g* for 5 min and resuspended in R10F medium.

African green monkey kidney epithelial Vero cells expressing human CD150 (Vero-CD150) (44) were grown in Dulbecco's modified Eagle's medium (DMEM; Lonza, Belgium) supplemented with 10% FBS (Sigma-Aldrich, USA), 100 IU of penicillin/ml and 100 μg of streptomycin/ml (Lonza, Belgium), and 2 mM l-glutamine (Lonza, Belgium).

### Viruses.

Recombinant MV strain Khartoum-Sudan (KS) expressing the fluorescent reporter protein Venus from an additional transcription unit in position 3 of the genome [rMV^KS^Venus(3)] is based on a wild-type virus isolated from the PBMC of a severe measles patient in Khartoum, Sudan (45). Virus stocks were generated in BLCL in R10F medium. Wild-type MV strain MVi/Amsterdam.NLD/19.11 (genotype D4) was isolated from a measles patient in Amsterdam, the Netherlands, in 2011. The virus stock was a passage 2 isolate grown in Vero-CD150 cells and had a titer of 1.6 × 10^6^ 50% tissue culture infectious dose (TCID_50_)/ml (the virus is available via the European Virus Archive). Endpoint titration on Vero-CD150 cells was performed to determine the virus titer, which was expressed as the number of TCID_50_ per 1 ml and calculated as described by Reed and Muench (46).

### MV infection.

Freshly isolated PBMC (2 × 10^6^ cells) were either cocultured with a low number (2 × 10^4^ cells) of autologous rMV^KS^Venus(3)-infected BLCL or inoculated with cell-free virus at an MOI of 1 in the presence of 10 μg/ml PHCSK_4_, an infection-enhancing synthetic lipopeptide. The BLCL coculture model is a dynamic system in which one MV-infected BLCL can infect multiple PBMC by direct cell-to-cell contact (27). The synthetic cationic lipopeptide PHCSK_4_ was previously shown to enhance MV binding to target cells (47). After 30 h, the infection levels of PBMC subsets were determined by flow cytometry.

As no autologous BLCL were available for the tonsil donors, tonsillar lymphocytes (≥5 × 10^5^ cells) were infected with cell-free virus at an MOI of 1 in the presence of infection-enhancing lipopeptide as described above. Infection levels were determined by flow cytometry after 24 h.

### Cell sorting.

PBMC isolated from buffy coats were enriched for B cells by partial depletion of CD14^+^ monocytes and CD3^+^ T cells using MicroBeads (Miltenyi Biotec). Subsequently, four lymphocyte subsets were sorted from the partially enriched PBMC or tonsils using a BD FACSAria II cell sorter (BD Biosciences): the remaining CD4^+^ naive and memory T cells and naive and memory B cells. Complete lymphocyte subset definitions are listed in Table 2. The sorted cells were inoculated with cell-free rMV^KS^Venus(3) at an MOI of 3 in the presence of infection-enhancing lipopeptide. Infection levels were determined by flow cytometry after 24 h, and the numbers of productively MV-infected cells were determined by infectious center assay.

### Flow cytometry.

T cells were divided into four main subsets: naive T cells, central memory T cells (T_CM_), CD45RA^−^ effector memory cells (T_EMRO_), and CD45RA^+^ effector memory cells (T_EMRA_). B cells were divided into four main subsets: naive B cells, non-class-switched IgM^+^ memory B cells, CD27^−^ IgM^−^ class-switched memory cells, and CD27^+^ IgM^−^ class-switched memory cells. Memory T and B cells were further subdivided into different specific subsets. Complete lymphocyte subset definitions are listed in Table 1. Flow cytometry was performed using a Fortessa cell analyzer or a BD FACSCanto II flow cytometer (BD Biosciences). The antibodies used in this experiment are listed in Table 3. The threshold for CD150 positivity was determined by using a fluorescein isothiocyanate (FITC)-conjugated isotype control: the histogram gate of the isotype background signal was set at 1% and this percentage was subsequently subtracted from the percentage of CD150^+^ cells. For sorted naive and memory CD4^+^ T and B cells, the cells were stained prior to sorting with the antibodies listed in Table 3. The infection levels were determined by detection of the Venus fluorescent signal or detection of intracellular MV NP within every lymphocyte subset. Cells infected with wild-type MV were fixed and permeabilized with a BD Cytofix/Cytoperm fixation and permeabilization kit according to the manufacturer's instructions (BD Biosciences), and the presence of MV NP was determined by intracellular staining with an FITC-conjugated monoclonal MV NP antibody (catalog number MAB8906F; Merck Millipore, USA). Infected cells were fixed with 2% paraformaldehyde prior to FACS measurement. Data were acquired with BD FACSDiva software and analyzed with FlowJo software.

**TABLE 3:** Flow cytometry antibody and cell-sorting antibody details

Assay and antigen	Antibody clone	Fluorochrome^*a*^	Manufacturer
Flow cytometry			
CD27	M-T271	BV421	BD Biosciences
IgM	MHM-88	BV510	BioLegend
CD38	HIT2	BV605	BioLegend
IgD	IA6-2	PerCP-Cy5.5	BioLegend
IgA	IS11-8E10	PE	Miltenyi Biotech
IgG	G18-145	PE-CF594	BD Biosciences
CD19	J3-119	PC7	Beckman Coulter
CD21	B-ly4	APC	BD Biosciences
CD24	ALB9	APC-AF750	Beckman Coulter
CD4	OKT4	BV510	BioLegend
CD45RA	HI100	BV605	BioLegend
CD28	CD28.2	PerCP-Cy5.5	BioLegend
CD3	SK7	PE	BD Biosciences
CCR7	150503	PE-CF594	BD Biosciences
TCRγδ	11F2	PE-Cy7	BD Biosciences
CXCR5	51505	APC	R&D Systems
CD8	SK1	APC-H7	BD Biosciences
CD25	BC96	BV421	BioLegend
CXCR3	1C6	BV711	BD Biosciences
CCR6	G034E3	PerCP-Cy5.5	BioLegend
CCR4	L291H4	PE-Cy7	BioLegend
CD127	A019D5	APC	BioLegend
CD150	A12	FITC	AbD Serotec
MV nucleoprotein	83KKII	FITC	Merck Millipore
Isotype	CLB-203	FITC	BD Biosciences
Cell sorting			
CD19	J3-119	PC7	Beckman Coulter
CD38	HIT-2	BV605	BioLegend
CD27	M-T271	BV421	BD Biosciences
IgM	MHM-88	BV510	BioLegend
CD3	UCHT1	PE	BD Biosciences
CCR7	150503	PE-CF594	BD Biosciences
CD8	SK1	APC-H7	BD Biosciences
CD4	RPA-T4	APC	BD Biosciences
CD45RO	UCHL1	FITC	eBioscience

a PerCP, peridinin chlorophyll protein; PE, phycoerythrin; APC, allophycocyanin; FITC, fluorescein isothiocyanate; BV, Brilliant violet; AF, Alexa Fluor.

### Infectious center assay.

Serial 2-fold dilutions of MV-infected sorted naive or memory lymphocytes were cocultured with BLCL (2 × 10^4^ cells/well) in a 96-well round-bottomed plate as described previously (48). The fluorescent signal was monitored by UV microscopy after 6 days. The numbers of MV-infected cells were calculated as 50% endpoints using the formula of Reed and Muench (46).

### Statistical analysis.

Differences between the percentages of CD150^+^ or infected cells were analyzed by a paired *t* test or the Wilcoxon signed-rank test.
